# The Photoreceptors Phototropin 1 and Phytochrome B Mediate Moonlight Perception and Response in *Arabidopsis thaliana*

**DOI:** 10.3390/plants15071041

**Published:** 2026-03-27

**Authors:** Sasank Sannidhi, Jeevan R. Singiri, Naveen Kumar Yarra, Nurit Novoplansky, Gideon Grafi

**Affiliations:** French Associates Institute for Agriculture and Biotechnology of Drylands, Jacob Blaustein Institutes for Desert Research, Ben-Gurion University of the Negev, Midreshet Ben Gurion 84990, Israel; sannidhisasank@gmail.com (S.S.); jeevannaveen01@gmail.com (J.R.S.); yarranaveenkumar61@gmail.com (N.K.Y.); nuritnov@bgu.ac.il (N.N.)

**Keywords:** moonlight perception, deetiolation, photoreceptors, phyB, phot1, phototropin mutants, *phyA* mutant, *phyB* mutant, nuclear morphology, *Arabidopsis thaliana*

## Abstract

Early and recent studies have demonstrated that exposure to moonlight influences the entire life cycle of plants from seed germination to vegetative growth and reproduction. Exposure to moonlight was found to induce genome reorganization in plants and significant changes in gene expression, protein, and metabolite profiles. However, the specific factors that facilitate moonlight perception are unknown. To uncover the photoreceptors responsible for moonlight perception, we analyzed *Arabidopsis* phototropin mutants (*phot1*, *phot2*, and *phot1phot2*) as well as the phytochrome mutants *phyA* and *phyB* for their response to full moonlight (FML). De-etiolation assays revealed that plants do perceive and respond to FML within 5 h of exposure. Thus, among the photoreceptor mutants analyzed, only *phot1* and *phot1phot2* were impaired in apical hook opening and cotyledon unfolding under FML. Interestingly, under high light intensity, all examined mutants underwent proper de-etiolation. Further analysis showed that *phot1* as well as *phyB* mutants were impaired in response to moonlight, displaying no changes in nuclear size and in protein profiles following exposure to FML and were comparable to plants exposed to dark. The FML (5 h exposure) did not induce the formation of fewer, large nuclear photobodies, as occurred following 5 h exposure to growth-room light. Our findings highlighted phot1 and phyB as photoreceptors necessary for plants to perceive and respond to FML. It is proposed that the initial perception of moonlight is facilitated by the blue-light receptor phot1 and is subsequently interpreted into a functional state by the R/FR receptor phyB.

## 1. Introduction

The moon orbits around the Earth in about 29.5 days. This period is known as the lunar cycle (also called the synodic cycle), which consists of four primary phases: the new moon, first quarter, full moon, and last quarter. The moon reflects sunlight essentially at a similar spectrum but at a very low intensity of about 0.32 lux (equating to 0.0059 mmol m^−2^ s^−1^) [1], which is significantly lower than sunlight intensity (~2000 mmol m^−2^ s^−1^) and below the minimal intensity required for photosynthesis (0.1 mmol m^−2^ s^−1^) [2]. In addition to its low intensity, moonlight exhibits notable spectral differences compared to sunlight; the red to far-red (R:FR) ratio of sunlight during the day exceeds 1.2, whereas the R:FR ratio of moonlight falls between 0.18 and 0.22 [3]. Notably, the distinctive characteristics of moonlight appear to be similar to the decrease in light intensity and R:FR ratio that occurs when plants shadow one another [4,5].

Traditional lunar farming constitutes a unique agricultural approach where planting, harvesting, and other agronomical activities are coordinated with the phases of the moon, with the aim of improving crop growth and enhancing yields [6,7]. However, this agricultural approach is often regarded as a myth, but recent studies have begun to uncover the molecular basis for plant response to moonlight and the moon’s impact on plant cell biology [6,8]. In *Coffea arabica*, Breitler et al. [3] presented strong evidence of transcriptional variation in response to full moonlight (FML), noting significant changes in the expression of clock genes, stress-response genes, and notably, the photoreceptor phot1. Additionally, Singiri et al. [9] showed that moonlight induces notable changes in the genome organization of tobacco and mustard plants, accompanied by alterations in DNA methylation and histone modifications, alongside changes in protein and metabolite profiles. Recently, experiments carried out on etiolated seedlings of Arabidopsis have shown that the opening of the apical hook and the unfolding of cotyledons take place after 5 h of exposure to FML, similar to their response to 5 h of light in a growth room (~150 mmol m^−2^ s^−1^) [10]. This indicates that, despite the moonlight’s low intensity, it is recognized as a signal by as-yet-unknown photoreceptors that trigger a noticeable response in the plants. 

Five photoreceptor classes have been characterized. The phytochrome group consists of five members in *Arabidopsis* designated phyA, phyB, phyC, phyD, and phyE, which perceive red/far-red light [11]. The cryptochrome group consists of three members, CRY1, CRY2, and CRY3 [12], and the phototropin group has two members, phot1 and phot2 [13,14]. Both the CRY and the phot groups perceive blue light [15]. In addition, there are three members of the LOV-containing F-box protein group [e.g., ZEITLUPE (ZTL), FLAVIN-BINDING, KELCH REPEAT, F-BOX 1 (FKF1), and LOV KELCH PROTEIN 2 (LKP2)] absorbing blue/UV-A light (320–500 nm) and a single photoreceptor, UVR8, which perceives UV-B light (280–320 nm) [15].

Studies in *Coffee arabica* and *Arabidopsis* showed that phot1 is upregulated following exposure to FML [3,10], while in tobacco, phyB and phot2-like proteins were upregulated [9]. Based on these findings, we selected phototropins and phytochromes for further study. Accordingly, we utilized mutants in phototropins (*phot1*, *phot2* and the double mutant *phot1phot2*) and in phytochromes (*phyA* and *phyB*) and identified phot1 and phyB as the main photoreceptors enabling plants to sense and respond to the moonlight.

## 2. Results

### 2.1. FML-Induced De-etiolation Is Impaired in phot1 and phot1phot2 Mutants

**Figure 1:**
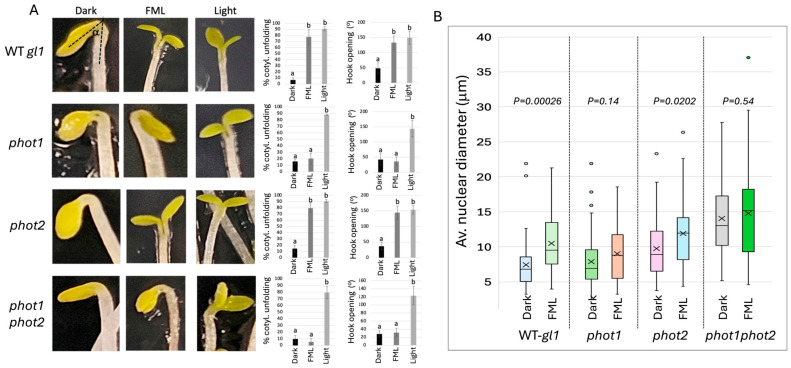
phot1 is required for the de-etiolation response under FML. (**A**) The response of WT *gl-1*, *phot1*, *phot2*, and *phot1phot2* etiolated seedlings to FML and growth-room light (light) as compared to dark (3 replicates, each 19–40 seedlings). The average percentage of cotyledon unfolding, and the average degree of hook opening (angle a) are shown to the right. Statistical analysis of the differences between the treatments shown in panel (**A**) was performed using one way ANOVA with Tukey HSD (Social Science Statistics). Bars represent the standard deviation. Different letters indicate statistically significant differences between treatments (*p* < 0.05). (**B**) No change in nuclear size in *phot1* and *phot1phot2* mutants. The leaves of the indicated *Arabidopsis* WT *gl-1* and phototropin mutants exposed to dark or to FML for 5 h were fixed in acetic acid:ethanol (1:3), and the nuclei were prepared, stained with DAPI, and visualized under a confocal microscope and the nuclear diameter was recorded. Boxplots representing nuclear diameter (n = 50) in the different lines following exposure to FML. *p*-value of differences between dark and FML treatments is indicated for each line (unpaired *t*-test, 2025 GraphPad software).

We first examined the capacity of Arabidopsis plants to respond to FML by analyzing etiolated seedlings for apical hook opening and cotyledon unfolding. These are characteristic responses of etiolated dicotyledonous seedlings stimulated within a few hours following exposure to white, fluorescent light [16]. To assess the role played by phototropins in apical hook opening and cotyledon unfolding, we examined the response of etiolated seedlings of WT *gl-1*, *phot1*, *phot2*, and the *phot1phot2* double mutant to 5 h exposure to FML in comparison to dark and growth-room (GR) light (light). The results showed (Figure 1A) that unlike WT *gl-1* and the *phot2* mutant, the *phot1* and *phot1phot2* mutants were impaired in cotyledon unfolding and apical hook opening following exposure to FML (Figure 1A). However, their reaction to GR light was indistinguishable from that of WT plants (Figure 1A), indicating that under conditions of high light intensity, phot1 is not required for de-etiolation, consistent with previous reports.

### 2.2. Plant Response to FML Requires phot1: Genome Organization

It has been shown previously that the size of nuclei derived from plants transitioning from dark to light or from plants exposed to FML is significantly increased, which might reflect genome reorganization accompanied by chromatin decondensation [9,17]. To identify the photoreceptors acting in moonlight perception, we examined changes in nuclear size following exposure to FML. Hence, 4-week-old seedlings of WT *gl-1* and the *phot* mutant lines—*phot1*, *phot2*, and the *phot1 phot2* double mutant—were subjected to dark or FML for 5 h; the leaves were collected, fixed in acetic acid/ethanol, and the nuclei were prepared, stained with DAPI, and inspected and measured under a confocal microscope. The results showed (Figure 1B) that exposure to FML induces a significant increase in the nuclear size of WT *gl-1* and the *phot2* mutant but not in the *phot1* or *phot1phot2* double mutant, suggesting that phot1 might be the principal blue-light receptor for moonlight perception. Interestingly, closer analysis of the results revealed that the *phot* mutants display a notable increase in nuclear size regardless of treatment, as compared to WT, which appeared to be an inherent feature of these mutants. Particularly, the double *phot1phot2* mutant displayed a 1.89-fold increase in nuclear diameter, a phenomenon which has not been described previously for *phot* mutants and will not be discussed further.

### 2.3. phot1 Is Required for Perception and Plant Response to Moonlight: Proteome Analysis

A comprehensive proteomic analysis was conducted to further explore the molecular responses of wild-type (WT *gl-1*) and *phot1phot2* mutant plants under full moonlight (FML) and dark conditions. Leaves from 4-week-old WT *gl-1* and *phot1phot2* mutant plants were harvested after 5 h of FML or dark exposure, and the proteins were extracted for proteomic profiling via mass spectrometry using the label-free quantification (LFQ) method.

In total, 2510 and 2569 proteins were cataloged as expressed in WT *gl-1* and the *pho1phot2* mutant, respectively, based on their presence in at least two replicates of one treatment group (Supplementary Tables S1 and S2).

**Figure 2:**
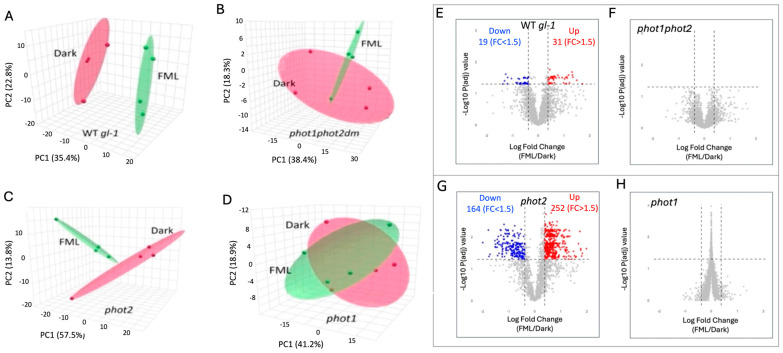
PCA score plots of expressed proteins in dark- and FML-treated wild-type (WT *gl-1*) (**A**), *phot1phot2dm* (**B**), *phot2* (**C**), and *phot1* (**D**) plants. Volcano plots demonstrating the fold change of proteins in FML- vs. dark-treated WT *gl-1* (**E**), the *phot1phot2* mutant (**F**), *phot2* (**G**) and the *phot1* mutant (**H**). Red and blue dots indicate upregulated and downregulated proteins, respectively, while gray dots indicate no change (FC > 1.5; *p* < 0.05). The numbers of upregulated and downregulated DEPs are shown at the top of panels (**E**,**G**). The threshold for the fold change (FC) is indicated. (*p* < 0.05).

**Figure 3:**
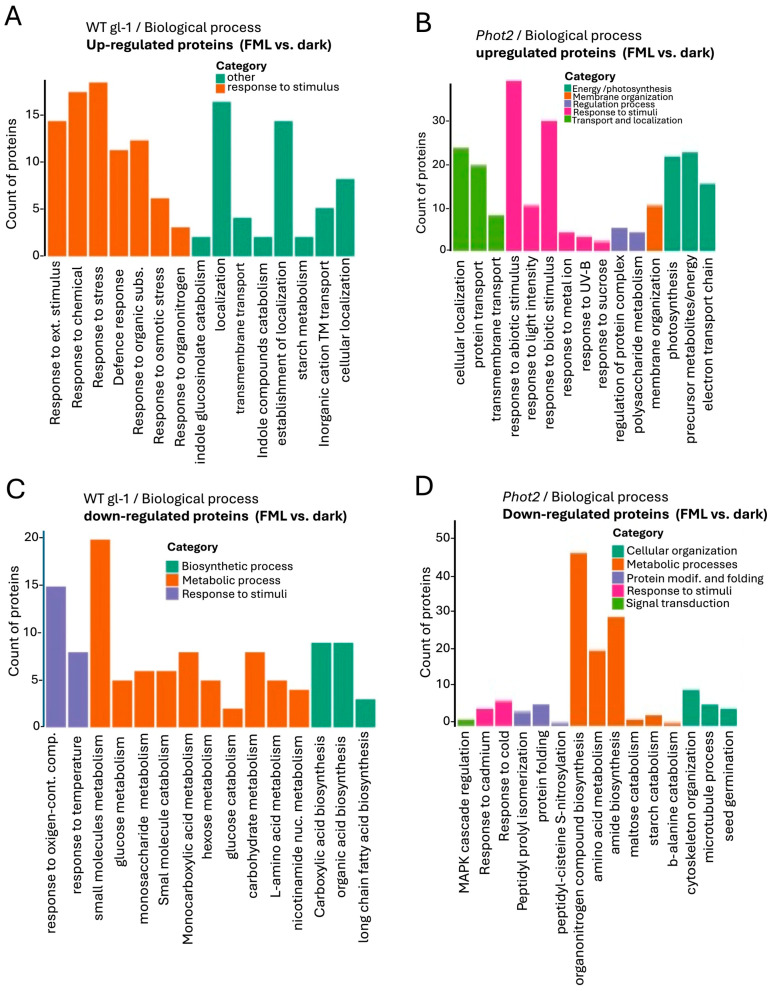
Categorization and bar chart representation of Gene Ontology term enrichment. Biological process category of upregulated (**A**,**C**) and downregulated (**B**,**D**) proteins in WT *gl-1* and *phot2* plants following exposure to FML as compared to dark. The Y-axis indicates the count of proteins associated with the term, while the X-axis lists the GO terms. Bars are color-coded according to the category of the enriched term, as indicated in the respective legends.

The PCA score plots revealed distinct clustering according to treatments in the WT *gl-1* plants (Figure 2A), but not in the *phot1phot2* mutant (Figure 2B). This is reflected in the volcano plots demonstrating the differentially expressed proteins (DEPs) in WT *gl-1* (Figure 2E) and no statistically significant DEPs in the *phot1phot2* mutant (Figure 2F). Using a cut-off of fold change FC > 1.5 and *p*-value < 0.05, a total of 50 proteins were identified as differentially expressed in WT *gl-1* plants (Supplementary Table S3), whereas no significant differential expression was detected in the *phot1phot2* mutant. Gene Ontology (GO) enrichment showed that in WT *gl-1* plants, under FML exposure, significant upregulation was observed in proteins associated with various biological processes (BPs), molecular functions (MFs), and cellular components (CCs). Within the BP category, upregulated proteins were particularly enriched in response to stimulus/stress, such as Cu-Zn superoxide dismutase 1 (Figure 3A). In the MF category, there was a notable enrichment of proteins involved in binding activities, including protein binding and ion binding (Figure S2A). Interestingly, categorization for CCs highlighted the vacuole as a prominent subcategory, with a substantial number of vacuolar-associated proteins showing upregulation under FML exposure (Supplementary Table S3 and Supplementary Figure S2C).

Conversely, downregulation was observed across multiple processes in WT *gl-1* plants under FML exposure. In the BP category, terms associated with metabolic functions, such as metabolic processes, carbohydrate-derivative metabolic processes, and organic acid biosynthetic processes showed notable downregulation (Figure 3B). In MF, subcategories like oxidoreductase activity, catalytic activity, and small-molecule binding were downregulated (Figure S2B), and for CCs, chloroplast stroma and plastid-associated proteins were prominent among the downregulated proteins (Supplementary Table S3, Figure S2D).

To further explore the individual contributions of phot1 and phot2, proteomic analyses were performed on *phot1* and *phot2* mutant plants after 5 h exposure to FML or dark. We identified 2132 and 2075 proteins cataloged as expressed in *phot1* and *phot2*, respectively (Supplementary Tables S4 and S5). The PCA plot for the *phot2* mutant showed separation between FML and dark (Supplementary Figure S2C) similar to WT *gl-1*, while the PCA plot for the *phot1* mutant showed no distinct separation (Figure 2D), reflecting an absence of molecular adjustments in response to FML exposure. Further statistical analysis revealed 416 proteins as differentially expressed with a cut-off FC > 1.5 and *p*-value < 0.05 in *phot2*, while none were identified as differentially expressed in *phot1*. Out of the DEPs, 252 were upregulated and 164 were downregulated in *phot2* upon exposure to FML vs. darkness (Supplementary Table S6). Volcano plots demonstrate the DEPs in *phot2* (Figure 2G) and the *phot1* mutant (Figure 2H).

In *phot2* under FML exposure, GO enrichment analysis showed upregulation in the BP category, similarly to WT *gl-1*, highlighting specifically the terms “response to abiotic and biotic stimulus” and, to a lesser extent, photosynthesis-related proteins, including the proton transport and electron transport chain (Figure 3C).

Within the MF category (Figure S3A), there was significant upregulation of proteins associated with binding activity and catalytic activity, including oxidoreductase activity and mRNA and protein binding. This is consistent with WT *gl-1* plants, which also showed enrichment in binding-related functions, particularly for proteins involved in signaling and environmental interactions. For the CC category (Figure S3C), enriched terms included the chloroplast thylakoid membrane, photosynthetic membrane, vacuole, chloroplast stroma, and plastid envelope. Notably, both WT *gl-1* and *phot2* exhibited a prominent presence of vacuolar-associated proteins (Supplementary Figure S2C and S3C).

Downregulated proteins in the BP category in *phot2* included (Figure 3D) terms related to carbohydrate metabolic processes, amino acid biosynthetic processes, and response to chemical stimulus, paralleling the downregulation in WT *gl-1* plants, which also showed reductions in metabolic activities. In the MF category (Figure S3B), downregulated proteins were associated with catalytic activity and binding activity, while within the CC category (Figure S3D), downregulated terms included the cytoplasm, anatomical structure and plastid (Supplementary Table S6), indicating decreased activity in these compartments under FML conditions.

### 2.4. Phytochrome B Is Required for Plant Response to Moonlight

**Figure 4:**
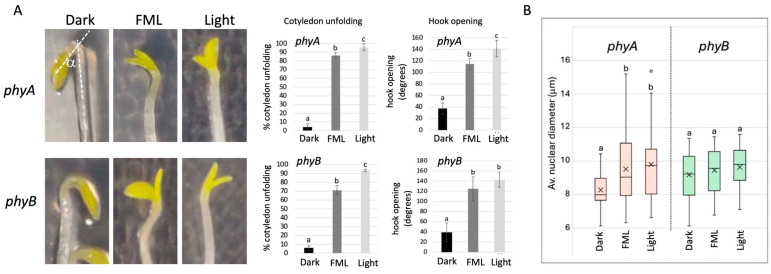
De-etiolation of *phyA* and *phyB* mutants. (**A**) The response of *phyA* and *phyB* etiolated seedlings to FML and growth-room light (light) as compared to dark. Data obtained from 3 replicates, each containing 19–40 etiolated seedlings. The average percentage of cotyledon unfolding, and the average degree of hook opening (angle a) are shown to the right. (**B**) Boxplots representing average nuclear diameter (n = 50) in *phyA* and *phyB* mutants following exposure to dark, FML and light. Statistical analysis of the differences between treatments shown in panels (**A**,**B**) was performed using one way ANOVA with Tukey HSD (Social Science Statistics). Bars represent the standard deviation. Different letters indicate statistically significant differences between treatments (*p* < 0.05).

PhyB plays a major role in sensing both low light intensity and reduction in the R:FR ratio (both are characteristics of moonlight), which commonly occur when plants are exposed to shade, triggering shade avoidance [18,19]. This function of phyB prompted us to investigate the role played by phytochromes in response to moonlight. Accordingly, we analyzed the de-etiolation capacity of the *phyA* and *phyB* mutants following exposure to FML and to growth-room light as compared to dark. Results showed (Figure 4A) that like WT plants [10], both *phyA* and *phyB* etiolated seedlings essentially responded to FML and to GR light by opening the apical hook and unfolding cotyledons, implying that phyA and phyB are not involved in initial moonlight perception.

The analysis of nuclear size showed (Figure 4B) that exposure to FML induces a significant increase in the nuclear size in the *phyA* mutant, similarly to WT *gl-1*, but not in the *phyB* mutant, implying that phyB might be involved in inducing nuclear reorganization following exposure to FML.

### 2.5. PhyB Is Required for Plant Response to Moonlight: Proteome Analysis

**Figure 5:**
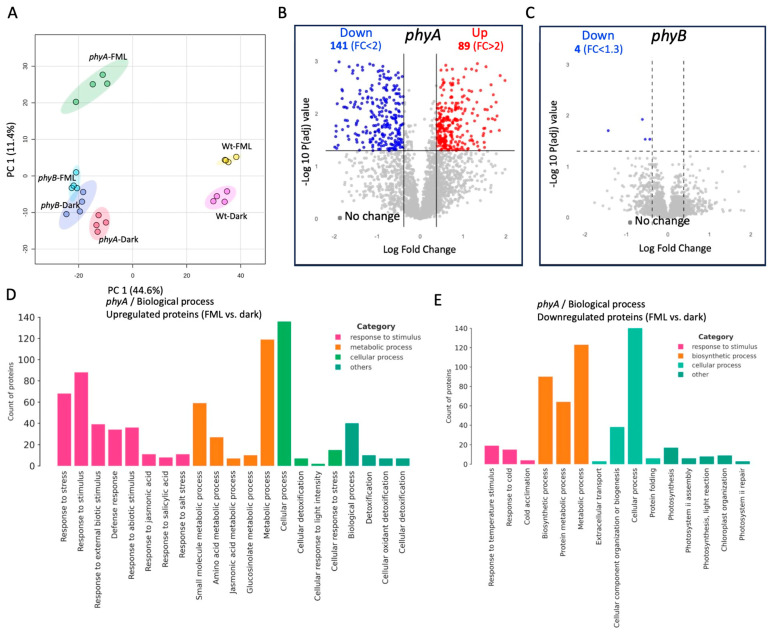
PhyB is required for plant response to FML. (**A**) PCA score plots of expressed proteins in dark- and FML-treated *phyA* and *phyB* mutant plants. (**B**,**C**) Volcano plots demonstrating the fold change of proteins under FML vs. dark in *phyA* (**B**) and *phyB* (**C**). Red and blue dots indicate upregulated and downregulated proteins, respectively. The numbers of upregulated and downregulated DEPs are shown at the top of panels (**B**,**C**). The threshold for the fold change (FC) is indicated. (*p* < 0.05). (**D**,**E**) Categorization by biological process of upregulated (**D**) and downregulated (**E**) proteins under FML versus dark in *phyA* mutant plants. Note the upregulation of a large number of stress-responsive proteins under FML.

We conducted proteome analysis to examine the response of *phyA* and *phyB* mutants to FML. After filtering out potential contaminants, including reverses (false positives from decoy databases) and those only identified by site, as well as filtering for proteins expressed in at least two replicates of at least one treatment group, 2867 expressed proteins were documented (Supplementary Table S7). Principal component analysis (PCA) separated the proteins according to treatment in *phyA,* while in the *phyB* mutant, proteins from the dark and FML conditions were clustered together (Figure 5A). This is illustrated in the volcano plots (Figure 5B,C), showing that *phyA* but not *phyB* responded to FML by changing their protein profiles. Among the 230 differentially expressed proteins (DEPs, FC >/< 2; *p* < 0.05) between the FML- and dark-treated *phyA* mutant, 89 and 141 proteins were upregulated and downregulated, respectively (Supplementary Table S8). Only four proteins in the *phyB* mutant exhibited a reduction of more than 1.3-fold in response to FML compared to dark conditions (Supplementary Table S9), indicating that at the protein level, the *phyB* mutant essentially perceives FML as equivalent to dark.

Similarly to WT plants (Figure 3) [10], categorization of the DEPs by biological process highlighted response to stimulus as the major subcategory upregulated following exposure of the *phyA* mutant to FML (Figure 5D,E). Within the molecular function category, the *phyA* mutant displayed a significant upregulation of proteins associated with small-molecule/ion binding activity (Figure S4A) and downregulation of nucleic acid-binding activity (Figure S4B). Interestingly, in WT *gl-1* and the *phot2* mutant, the categorization for MF revealed an opposite trend, highlighting an upregulation of mRNA and protein binding activity (Figures S2A and S3A), alongside a downregulation of small-molecule/ion binding activity (Figures S2B and S3B).

### 2.6. Growth-Room Light but Not FML Triggers the Accumulation of phyB-GFP in Large Nuclear Photobodies

**Figure 6:**
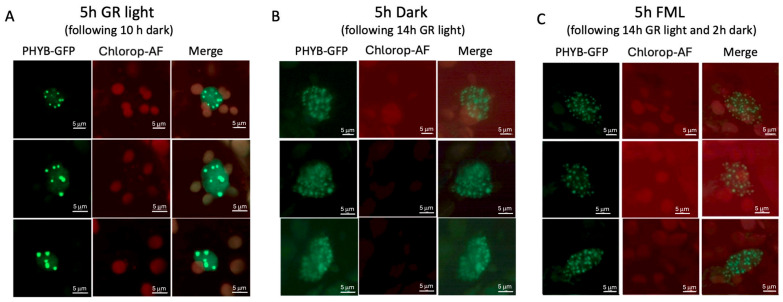
PhyB-GFP large photobodies (PBs) disassemble in the dark and are not recovered following exposure to FML. (**A**) Following 10 h in dark, phyB-GFP-expressing plants were subjected to GR light for 5 h. Representative confocal images are shown where phyB-GFP is assembled into fewer large, intensely fluorescing PBs. (**B**) Following 14 h in light, plants were subjected to 7 h of darkness, demonstrating disassembly of large PBs into small, dispersed, and faintly fluorescing PBs. (**C**) Exposure to 5 h FML, following 14 h GR light and 2 h dark. Note, examples of three nuclei are presented for each treatment. Chloro-AF, Chloroplast AutoFluorescence in red.

It has been previously shown that phyB photobodies undergo two major transitions: the first occurs after 12 h in darkness, where large-sized photobodies disassemble into many small photobodies, while the second occurs after 18 h of exposure to darkness, whereby PBs are completely lost and phyB shows even distribution in the nucleus [20]. We wanted to examine the capacity of the FML to induce the formation of large phyB-GFP PBs. To this end, plants expressing phyB-GFP [21] were subjected to 10 h of darkness followed by 5 h of GR light (~155 mmol m^−2^ s^−1^; R:FR = 10.5, Supplementary Figure S1). Plants were also exposed to 7 h of darkness or to 2 h darkness followed by 5 h of FML after being subjected to 14 h of GR light. Inspection under a confocal microscope showed that after a 5 h exposure to GR light, the plants exhibited a lower number of large and highly fluorescent PBs within the nucleus (Figure 6A). Following a subsequent 7 h period of darkness, these large PBs were broken down into numerous small, somewhat dispersed, and faintly fluorescing PBs (Figure 6B), as previously documented [20]. Following exposure to FML, the appearance of nuclear PBs was not changed and was essentially similar to those displayed by plants under 7 h of dark (Figure 6C). During this period, we could not detect a phyB-GFP signal outside the nucleus.

## 3. Discussion

The influence of the moon on living organisms is often emphasized through its physical characteristics related to gravitational and electromagnetic effects. Recent studies have highlighted moonlight as a signaling entity that provokes stress-like responses characterized by changes in genome organization and gene expression [3,9,10,22]. Although the moon reflects the sunlight at a very low intensity, this light is sufficient to induce biological reactions in plants. This is demonstrated by the capacity of moonlight to bring about photomorphogenic responses in etiolated *Arabidopsis* seedlings, that is, apical hook opening and cotyledon unfolding. We showed by using phototropin and phytochrome mutants, that phot1 and phyB are probably the major receptors required for moonlight perception and response by plants.

Phototropins are plant-specific blue-light receptors implicated in phototropism, chloroplast movement, leaf expansion, and stomatal opening [23]. These processes contribute to the optimization of photosynthesis by enhancing the efficient capture of light energy, reducing photodamage, and facilitating the acquisition of CO_2_. Both phot1 and phot2 have distinct roles in light perception, with phot1 particularly responding to low blue-light intensity, while phot2 is less sensitive to low light and operates under a relatively high blue-light intensity [24,25]. Our data are consistent with the roles of phototropins, showing that de-etiolation under FML (~0.0055 mmol m^−2^ s^−1^) was impaired in the absence of a functional phot1 but not in the absence of phot2. Interestingly, under conditions of high light intensity, de-etiolation occurs effectively even in the absence of phot1. This indicates that other photoreceptors, most likely phot2, function under high light intensity to facilitate de-etiolation in the absence of phot1 [25].

Consistent with previous reports [3,9,10], the response of WT and mutant plants (*phyA*, *phot2*) to FML highlighted the upregulation of proteins responding to stimulus, further supporting the notion that moonlight is perceived by plants as a stress signal. If FML is perceived by plants as a stress signal, what is then the actual stress imposed by the light of the full moon? Obviously, the moon reflects the sunlight at an extremely low intensity that is far below the intensity required to drive photosynthesis. Furthermore, although the moon reflects sunlight at an essentially comparable spectrum, there exist spectral differences between sunlight and moonlight. Thus, the red to far-red (R:FR) ratio of sunlight exceeds 1.2, while the R:FR ratio of moonlight varies from 0.18 to 0.22 [3], and thus the moonlight has about 5-fold less red-light intensity as compared to far-red light. This R:FR ratio of the moonlight is reminiscent of the low R:FR ratio when plants are grown under the canopy of other plants and exposed to shade [26]. Indeed, shade can trigger a stress response associated with ethylene biosynthesis, acceleretaed senescence, as well as an increase in reactive oxygen species and nitric oxide [27,28]. The reduction in the R:FR ratio is sensed by phyB, which plays a major role in triggering shade avoidance [18]. Thus, similarly to the effect of shade, the low R:FR ratio of the FML might be interpreted as shade stress that induces a stress response, which is accompanied by the upregulation of stress-related proteins.

Consistent with previous reports, phyB-GFP is clustered in fewer, large nuclear PBs when plants are exposed to high GR light (~155 mmol m^−2^ s^−1^) for 5 h. These PBs are broken into many small, dispersed PBs in the dark, which are retained in the nucleus, at least after 5 h in the dark. Exposure to FML did not induce the formation of large, fewer PBs, but the many small PBs remain structurally defined and are not dispersed as they are under dark conditions. It seems that the FML slows down the dispersion of the small PBs that would otherwise take place under dark conditions. Previous reports demonstrated that under high red-light intensity, which promotes conversion to Pfr, phyB is localized to fewer nuclear PBs. However, under darkness or a low R:FR ratio, phyB is found in many smaller nuclear photobodies or dispersed in the nucleoplasm [29]. Also, reducing irradiance while maintaining the R:FR ratio resulted in the formation of small nuclear bodies that contain phyB, suggesting that phyB can sense and respond to both low light intensity and a low R:FR ratio [19]. Despite the conversion to the Pr form occurring in darkness or under FML, we were unable to observe phyB-GFP outside the nucleus.

Taken together, these results indicate that the initial perception of moonlight is mediated by the blue-light receptor phot1 and is subsequently interpreted into a functional response by the R/FR receptor phyB. However, our data does not exclude the possibility of a cross-talk between photorecptors, particularly the blue-light/UV-A receptors and cryptochromes (CRYs), in the perception and response of plants to moonlight. Accordingly, a phytochrome–cryptochrome coaction has been demonstrated through physical interaction between phyB and CRY1 or phyB and CRY2 [30,31]. Both photoreceptors also interact with other regulatory proteins such as the PHYTOCHROME INTERACTING FACTORS (PIFs) and the CONSTITUTIVE PHOTOMORPHOGENIC 1/SUPPRESSOR OF PHYA-105 (COP1/SPA1) E3 ubiquitin ligase complex to control transcription [32]. In addition, both appear to control chromatin organization in response to low light in *Arabidopsis* [33]. The spectral characteristics of full moonlight, namely its low intensity and low R:FR ratio, could be interpreted by plants as a shade-like signal, which is accompanied by the induction of stress-associated genes. Under such conditions, phyB is expected to accumulate predominantly in the Pr form, resulting in alterations to its nuclear organization from fewer, large photobodies to a larger number of smaller photobodies, where it may exert specific nuclear functions. In line with this view, overexpression of Pr-phyB has been shown to suppress photomorphogenesis under far-red light, at least in part through the stabilization of the COP1–SPA1 E3 ubiquitin ligase in the nucleus [34]; the COP1-SPA1 E3 ligase ubiquitinates light-responsive transcription factors and directs them for degradation [35].

## 4. Materials and Methods

### 4.1. Plant Growth and Exposure to FML

The *Arabidopsis* WT *gl-1 and* the *phot* mutant lines *phot1*, *phot2*, and *phot1-phot2 dm*, all mutants in wild-type *Arabidopsis thaliana* (*gl-1*, ecotype Columbia), were obtained from Stuart Sullivan and John M. Christie [36]. The phytochrome *phyA-201* mutant (in Ler background) [37], and *phyB-9* and phyB-GFP (in Col-0 background) [21] were obtained from Ferenc Nagy. Seeds were sown in pots containing standard gardening soil composed of peat and perlite (2:1 ratio) and grown in a growth-room under 65–70% humidity, 24 °C ± 1 °C temperature, and 14/10 h (day/night) photoperiod conditions. The sunlight and growth-room light intensity and spectral measurements shown in Figure S1 (supplementary figures) were taken by a Li-Cor LI-180 Spectrometer (LICORbio, Lincoln, NE, USA). At full-moon stage (when the moon is half way to zenith), seedlings at the four-leaf stage were moved from the growth room after being in dark for 2 h and exposed for 5 h to FML or to 5 h dark; dark-treated plants were kept next to the FML-exposed plants by covering them with black cloth. The seedlings of the phototropin mutants were exposed to FML during June 2023, whereas those of phytochrome mutants were exposed during August 2023. The seedlings were collected and frozen immediately in liquid nitrogen and kept at −80 °C, or fixed in acetic acid/ethanol (1:3 *v*/*v*) and stored at −20 °C, until further use.

### 4.2. Response of Etiolated Seedlings to FML

The Arabidopsis thaliana WT, phot, and phy mutant seeds were surface-sterilized with 70% EtOH for 2 min followed by 50% bleach for 5–10 min. Seeds were washed with sterile H_2_O and planted in Petri dishes containing MS growth medium [38] and 0.8% agar. Seeds were incubated for 2 to 3 days at 4 °C, then exposed to red light for 1 h to induce uniform germination [16]. The seedlings were incubated in the dark at 23–25 °C for 4 days to produce etiolated seedlings. De-etiolation experiments were performed for each line in 3 replicates, each containing between 19 and 40 seedlings. Seedlings were exposed to FML or to growth-room light for 5 h, and the number of seedlings showing cotyledon unfolding and the degree of apical hook opening were recorded. Statistical significance between the treatments was determined using a one-way ANOVA with Tukey HSD (Social Science Statistics).

### 4.3. Nuclei Isolation and Confocal Microscope Inspection

Nuclei were prepared from fixed leaf samples of WT and the mutants essentially as described by Saxena et al. [39]. Briefly, leaves were chopped using a razor blade in nuclei isolation buffer (NIB) (10 mM MES-KOH, pH 5.5, 0.2 M sucrose, 2.5 mM EDTA, 2.5 mM dithiothreitol, 0.1 mM spermine, 10 mM NaCl, 10 mM KCl, 0.15% Triton X-100). The homogenate is gently stirred for 45 min at 4 °C and filtered through 100 μm nylon mesh followed by 30 μm nylon mesh. The filtered extract is centrifuged for 8 min at 2000 RPM at 4 °C. The pellet was gently washed to remove the upper chloroplast layer and the nuclei pellets were recovered and washed twice with NIB. The nuclei were fixed in ethanol:acetic acid (3:1 *v*/*v*) and stored at −20 °C until further used. The nuclei were stained for 10 min with 10 μg/mL diamidino-phenyl-indole (DAPI), washed twice with 2× SSC (300 mM NaCl, 30 mM Na-citrate, pH 7.0), and mounted in Vectashield (Vector Laboratories, Burlingame, CA, USA). The nuclei size measurements were done using a confocal microscope (Zeiss LSM 900, Carl Zeiss AG, Oberkochen, Germany) and the data was processed using Excel software (Microsoft, Redmond, WA, USA).

### 4.4. Proteome Analysis

For proteomic analysis, 4 replicates of 10 mg of ground leaves derived from 5 h dark- and 5 h FML-treated plants were placed in 2 mL tubes and incubated with 100 µL of NETN buffer (100 mM NaCl, 1 mM EDTA, 20 mM Tris, pH 8.0, and 0.5% NP-40) at 4 °C for 6 h with gentle rotation, then centrifuged at 4 °C at high speed for 10 min. A total of 50 µL of the supernatants was collected and stored at −20 °C until used for comparative, quantitative proteome analysis. Proteome analysis was performed by the proteomic services of the Smoler Protein Research Centre at the Technion, Haifa, Israel, using LC-MS/MS on LTQ Orbitrap (ThermoFisher Scientific, Waltham, MA, USA). Protein identification and quantification were done using MaxQuant, using *Arabidopsis thaliana* proteins from UniProt as a reference. Quantification and normalization were performed using the LFQ method and subsequent bioinformatics analysis by using Perseus software v4.1a [40]. Proteins marked as “contaminant” and “only identified by site” were filtered out. In an additional step, only proteins in which at least one of the groups has at least 2 non-zero replicates, proteins having at least 2 peptides, and a peptide sequence coverage of more than 20% were retained. A protein is considered differentially expressed if it has a nominal *p*-value < 0.05 and an absolute fold change > 1.5.

### 4.5. Nuclear Size Measurements

The nuclei were inspected under a confocal microscope (Zeiss LSM 900) and nuclear size measurements were performed on images processed using ZEN Blue Software 3.0 (Zeiss). The processed images were analyzed for nuclear size and the measurements were exported to Microsoft Excel for further statistical analysis. Student’s *t*-test was applied to compare the nuclear sizes between different experimental conditions, with the statistical significance set at *p* < 0.05. The average nuclear sizes were calculated and reported alongside the standard deviations to summarize the data.

### 4.6. Gene Ontology (GO) Analysis

GO analysis was conducted to explore the biological processes, molecular functions, and cellular components enriched in the proteomic data. GO terms were identified using the gProfiler online tool (https://biit.cs.ut.ee/gprofiler/gost, accessed on 24 February 2026) and the Bingo plugin in Cystoscope [41]. Bar plots representing the enrichment of GO terms were generated using R software 2025.09.2, employing the ggplot2 package for visual representation of the results.

## Data Availability

The original contributions presented in this study are included in the article/Supplementary Material. Further inquiries can be directed to the corresponding author.

## Supplementary Materials

The following supporting information can be downloaded at https://www.mdpi.com/article/10.3390/plants15071041/s1, Table S1. List of expressed proteins in WT gl-1 plants following 5 h exposure to FML and Dark; Table S2. List of expressed proteins in phot1phot2dm plants in Dark and FML using perseus; Table S3. List of significantly differentially expressed proteins in WT gl-1 FML vs Dark (padj < 0.05 and FCabs > 1.5); Table S4. List of expressed proteins in phot1 plants Dark and FML using perseus; Table S5. List of expressed proteins in phot2 mutant following 5 h exposure to FML and Dark; Table S6. List of significantly differentially expressed proteins FML vs. Dark in phot2 (padj < 0.05 and Fold change abs > 1.5); Table S7. List of expressed proteins present in both FML and Dark-treated plants of Arabidopsis thaliana mutants phyA and phyB; Table S8. List of differentially expressed proteins in FML exposed plants when compared to Dark in Arabidopsis phyA mutant (FC > 2; *p*-value < 0.05); Table S9. List of differentially expressed proteins in FML exposed plants when compared to Dark in Arabidopsis phyB mutant (FC > 1.5; *p*-value < 0.05); Figure S1. The intensity and spectral measurements of sun light and growth room light; Figure S2. Categorization and bar chart representation of gene ontology term enrichment of WT gl-1 DEPs between FML and dark; Figure S3. Categorization and bar chart representation of gene ontology term enrichment of phot2 DEPs between FML and dark; Figure S4. Categorization and bar chart representation of gene ontology term enrichment of phyA DEPs between FML and dark.
