# Supplementary material for: The Photoreceptors Phototropin 1 and Phytochrome B Mediate Moonlight Perception and Response in Arabidopsis thaliana
Source: Plants (Basel). 2026 Mar 27;15(7):1041. doi: 10.3390/plants15071041 (PMC13075113; doi:10.3390/plants15071041)
Supplement: Supplementary file 1 [file plants-15-01041-s001.zip › Supplementary figures.pdf]

Supplementary materials

The photoreceptors phototropin 1 and phytochrome B mediate moonlight perception and response in *Arabidopsis thaliana*

Sasank Sannidhi[†], Jeevan R. Singiri[†], Naveen Kumar Yarra, Nurit Novoplansky, and Gideon Grafi[1]

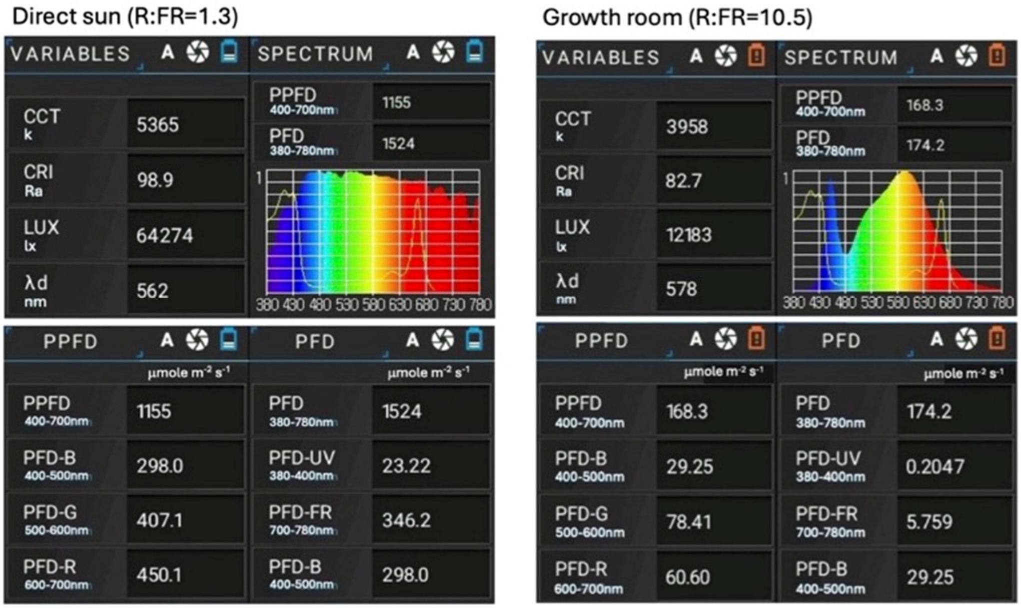

Figure S1. The intensity and spectral measurements of sun light and growth room light. Measurements were performed by LICOR 180. The R:FR ratio is given in the brackets. Note, the intensity and spectrum of FML cannot be taken by LICORE 180 as the reported FML intensity (~ 0.32 lx, Kyba et al., 2017) is below the measurement range of the device (70-150,000 lux https://www.licor.com/products/light/spectrometer).

.

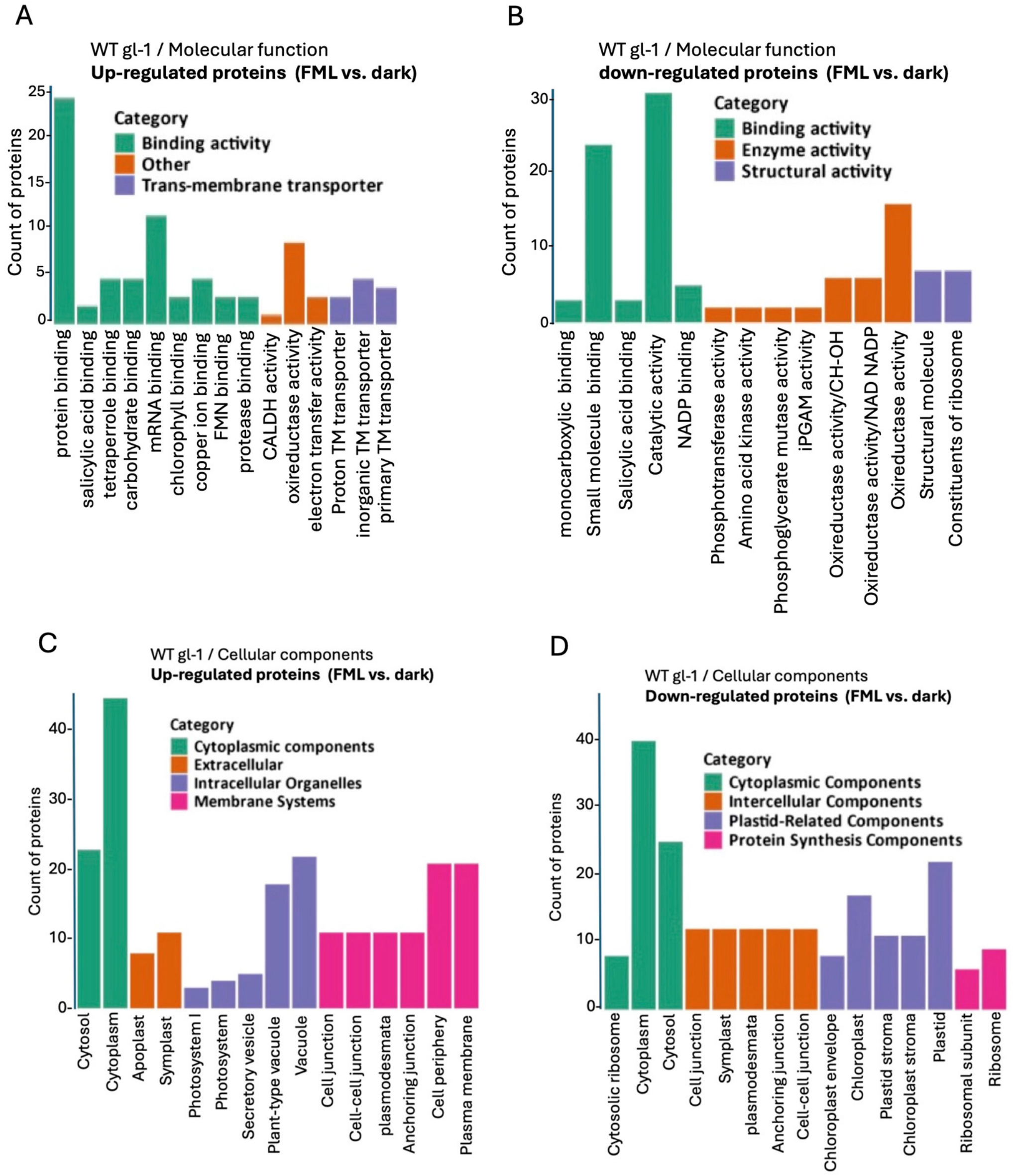

Figure S2. Categorization and bar chart representation of gene ontology term enrichment of WT gl-1 DEPs between FML and dark. Molecular function category of upregulated (A) and down-regulated (B) proteins and cellular component category of upregulated (C) and down-regulated (D) proteins in WT *gl-1* plants following exposure to FML as compared to dark. The Y-axis indicates the count of proteins associated with the term, while the X-axis lists the GO terms. Bars are color-coded according to the category of enriched term, as indicated in the respective legends.

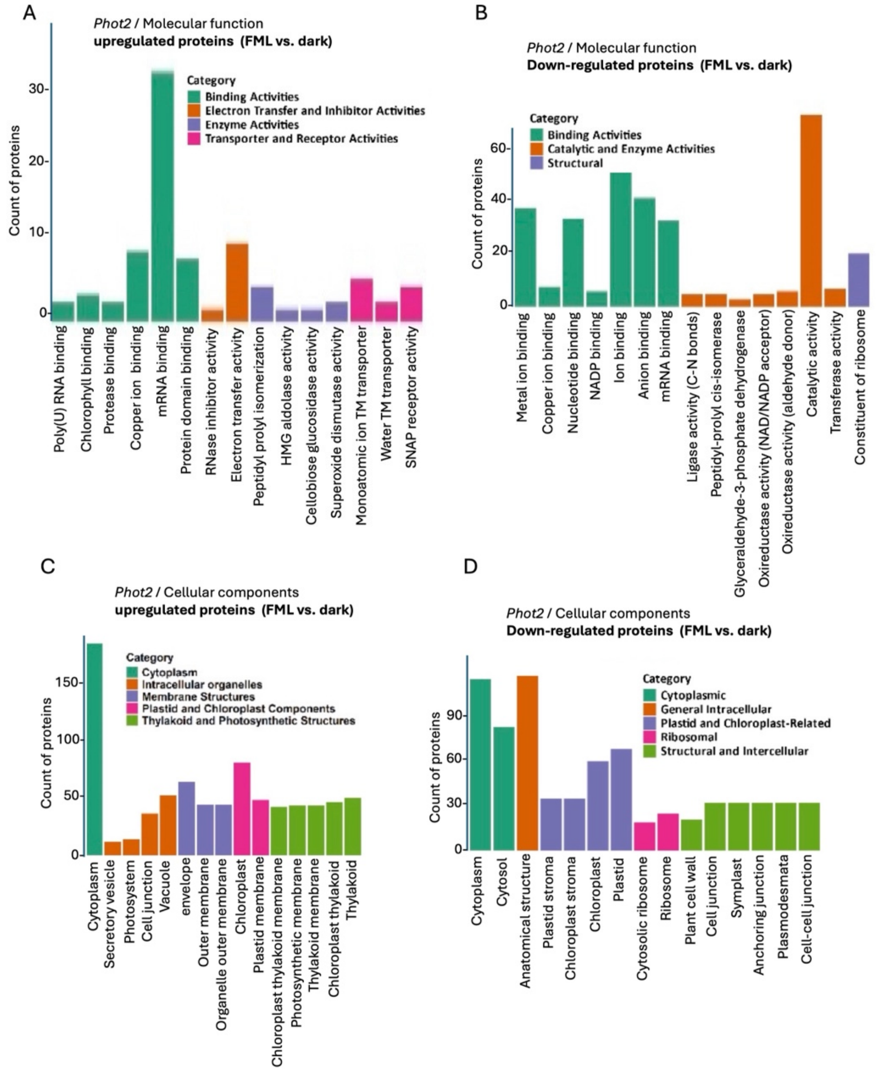

Figure S3. Categorization and bar chart representation of gene ontology term enrichment of *phot2* DEPs between FML and dark. Molecular function category of upregulated (A) and down-regulated (B) proteins and cellular component category of upregulated (C) and down-regulated (D) proteins in *phot2* following exposure to FML as compared to dark. The Y-axis indicates the count of proteins associated with the term, while the X-axis lists the GO terms. Bars are color-coded according to the category of enriched term, as indicated in the respective legends.

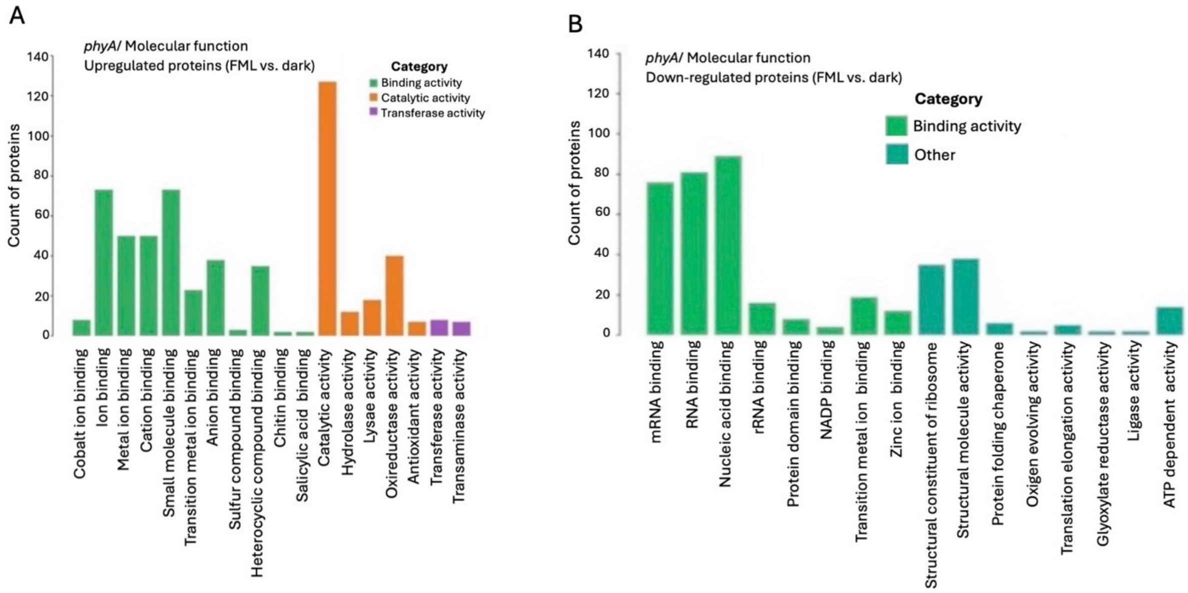

Figure S4. Categorization and bar chart representation of gene ontology term enrichment of *phyA* DEPs between FML and dark. Molecular function category of upregulated (A) and down-regulated (B) proteins in *phyA* mutant plants following exposure to FML as compared to dark. The Y-axis indicates the count of proteins associated with the term, while the X-axis lists the GO terms. Bars are color-coded according to the category of enriched term, as indicated in the respective legends.
